# Analysis of Hepatitis B Virus Genotype D in Greenland Suggests the Presence of a Novel Quasi-Subgenotype

**DOI:** 10.3389/fmicb.2020.602296

**Published:** 2021-01-15

**Authors:** Adriano de Bernardi Schneider, Carla Osiowy, Reilly Hostager, Henrik Krarup, Malene Børresen, Yasuhito Tanaka, Taylor Morriseau, Joel O. Wertheim

**Affiliations:** ^1^Department of Medicine, University of California San Diego, San Diego, CA, United States; ^2^National Microbiology Laboratory, Public Health Agency of Canada, Winnipeg, MB, Canada; ^3^Department of Molecular Diagnostics, Aalborg University Hospital, Aalborg, Denmark; ^4^Department of Medical Gastroenterology, Aalborg University Hospital, Aalborg, Denmark; ^5^Clinical Institute, Aalborg University, Aalborg, Denmark; ^6^Department of Epidemiological Research, Statens Serum Institut, Copenhagen, Denmark; ^7^Department of Virology & Liver, Nagoya City University Graduate School of Medical Sciences, Nagoya, Japan

**Keywords:** evolution, HBV, phylogenetics, phylogenomics, hepatitis

## Abstract

A disproportionate number of Greenland's Inuit population are chronically infected with Hepatitis B virus (HBV; 5–10%). HBV genotypes B and D are most prevalent in the circumpolar Arctic. Here, we report 39 novel HBV/D sequences from individuals residing in southwestern Greenland. We performed phylodynamic analyses with ancient HBV DNA calibrators to investigate the origin and relationship of these taxa to other HBV sequences. We inferred a substitution rate of 1.4 × 10^−5^ [95% HPD 8.8 × 10^−6^, 2.0 × 10^−5^] and a time to the most recent common ancestor of 629 CE [95% HPD 37–1138 CE]. The Greenland taxa form a sister clade to HBV/D2 sequences, specifically New Caledonian and Indigenous Taiwanese sequences. The Greenland sequences share amino acid signatures with subgenotypes D1 and D2 and ~97% sequence identity. Our results suggest the classification of these novel sequences does not fit within the current nomenclature. Thus, we propose these taxa be considered a novel quasi-subgenotype.

## 1. Introduction

Hepatitis B virus (HBV) is an ancient virus that remains a substantial public health burden. Transmitted by bodily fluid, it is one of the most prevalent viruses in the world, infecting over 2 billion people, 257 million of whom are chronically infected (WHO, 2019).

HBV is classified into nine distinct genotypes (A through I) and a putative 10th genotype (J) (Kramvis, 2014). Precedent sorting of genotype identity is calculated by a distance-based sequence threshold of 8% (Okamoto et al., 1988; Zhou and Holmes, 2007; Locarnini et al., 2013; Araujo, 2015; Lin and Kao, 2015; Littlejohn et al., 2016). Subgenotypes are traditionally assigned based on a 4–7.5% threshold across the full genome (McNaughton et al., 2020), though new phylogenetic inference methods to determine subgenotype have been suggested (Yin et al., 2019). McNaughton et al. (2020) notes that all genotypes have subgenotype sequence divergence rates of 3–8%, with the exception of genotype D, which is 2–4%, indicating a lack of long evolutionary branches separating well-defined clades. Recent suggestions for HBV taxonomic nomenclature include assigning the term “quasi-subgenotype” to a lineage that does not meet the criteria nor clearly belongs to a defined subgenotype based on <4% nucleotide divergence among complete genomes following detailed phylogenetic analysis (Pourkarim et al., 2010).

HBV was considered hyperendemic in the western circumpolar Arctic (Alaska, Canada, and Greenland) before the introduction of HBV vaccination programs (Lavanchy, 2008) with the burden of viral hepatitis disproportionately borne by Indigenous populations. Prior to the introduction of HBV vaccination programs in the Canadian Arctic in the 1990s, HBV prevalence was ~3% among Inuit populations (Osiowy et al., 2013); moreover, the risk of lifetime exposure to HBV was 25% or five times higher than the non-Indigenous population (Tulisov et al., 2007). Approximately 5–10% of Greenland's Inuit population remains chronically infected with HBV (Børresen et al., 2011; Rex et al., 2015). Since 1965, serosurveys of HBV exposure among Greenland residents have demonstrated high rates of exposure among adults and chronic infection rates 14–40 times higher than the US and northern Europe (Tulisov et al., 2007). Genotypes B and D predominate in the North American circumpolar Arctic (Osiowy et al., 2013). Western Greenland's infections are represented by two genotypes: a majority genotype D and a minority genotype A (Osiowy et al., 2013). While infancy is regarded as the most receptive stage of HBV infection, the incidence of active infection increases in adolescence (Tulisov et al., 2007). This highlights transmission by sexual contact, which is the primary method for HIV transmission in Greenland (Bjorn-Mortensen et al., 2013).

The most prevalent and endemic subgenotype, B5 (formerly B6), within the Alaska Native and Inuit populations of the western circumpolar Arctic may have originated coincident with the rapid movement of the Thule from regions of Alaska to the eastern Arctic (Bouckaert et al., 2017) ~1,000 years before present (YBP). Greenland Inuit harboring genotype D HBV (HBV/D) infections, such as those discussed in this study, may represent another endemic lineage with high rates of chronic infection and, importantly, a unique coalescent origin from other defined HBV/D subgenotypes. Known presence of HBV/D in the Arctic is limited to Greenland (Langer et al., 1997), Western Canada, and Alaska (Livingston et al., 2007; Osiowy et al., 2011). Association of HBV/D with First Nation Dene living in the western Arctic has also been observed, suggesting a separate introduction from Greenlandic Inuit (Osiowy et al., 2011).

Additionally, Inuit residing in the Canadian Arctic infected with subgenotype HBV/B5 experience less severe clinical outcomes typical of non-Indigenous people infected with other HBV/B subgenotypes (Minuk and Uhanova, 2003). This reduced risk phenomenon for Inuit infected with HBV/D is unknown. Historically, genotype D has poorer clinical prognoses and a lower response to therapy (McMahon, 2008; Yin et al., 2019). Specific basal core promoter (BCP) nucleotide mutations, T1762 and A1764, are important markers associated with liver disease progression and development of hepatocellular carcinoma (HCC) (Yang et al., 2016), and they are highly associated with HBV/D (Lin and Kao, 2015).

Despite its broad geographical distribution and large volume of sequence data, the evolutionary history of HBV remains ambiguous. The difference in substitution rates depending on whether the infection is acute, chronic, or within-vs.-between hosts (Vrancken et al., 2014) is compounded by discrepant calibrations (i.e., fossil-based or tip-dated). Ancient DNA (aDNA) calibration for exogenous viruses has been shown to tease apart some of the evolutionary mystery of HBV (Mühlemann et al., 2018), revealing a surprising lack of temporal genetic change in the last half millennium (Krause-Kyora et al., 2018). HBV is a suitable candidate for aDNA external calibration because of its high viremic levels, even during periods of prolonged infection, and covalently closed circular DNA (cccDNA) genome structure (Kramvis, 2014).

In this study, we report 39 novel Greenland taxa and investigate their phylogenetic relationships and the time to the most recent common ancestor (TMRCA) of HBV/D in Greenland. To understand how and when HBV reached Inuit populations, we analyzed novel sequence data combined with publicly available datasets and ancestral sequences. Here, we investigate the Greenland clade's genetic composition and existing genotype D heterogeneity and present the description of a novel quasi-subgenotype.

## 2. Methods

### 2.1. Data Collection

**Figure 1:**
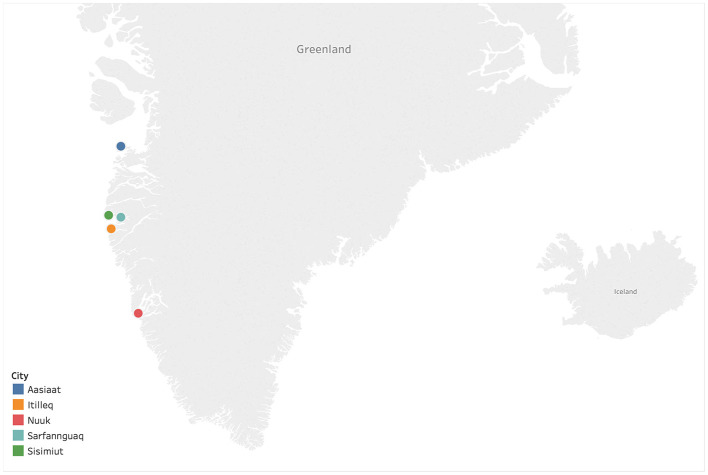
Sampling locations of Hepatitis B virus (HBV) isolates within Western Greenland. The map was generated using Tableau Desktop 2009.1 (https://www.tableau.com).

Thirty-nine serum specimens were collected from 25 individuals infected with HBV from five settlements in Western Greenland: Aasiaat, Itilleq, Nuuk, Sarfannguaq, and Sisimiut (Figure 1), as previously described (Kowalec et al., 2013; Bouckaert et al., 2017) (Supplementary Table 1). In certain cases, consecutive paired sera separated 5–10 years were collected from individuals for the purpose of investigating the intrapatient genetic diversity of HBV (Kowalec et al., 2013). All specimens were collected on dates ranging from 1998 to 2017 (Supplementary Table 1). Viral genomic DNA was extracted from 200 μL sera and amplified as previously described (Bouckaert et al., 2017). Amplicons were sequenced with an AB 3730 XL DNA analyzer using Big Dye 3.1 terminator chemistry (Thermo Fisher Scientific, Burlington, ON, Canada). Sequences were assembled and analyzed using DNA sequence analysis software (Lasergene software suite v 15.0.0, DNASTAR, Madison, WI, USA).

### 2.2. Taxon Sampling

We created two datasets for the analysis of the Greenland taxa. The first dataset (dataset 1) was 3,567 nucleotides in length and included genomes larger than 2,900 nucleotides for a total of 1,921 sequences from all HBV genotypes. This dataset was created in order to assess the clustering of the Greenland taxa within existing HBV diversity. Dataset 1 was subsampled to contain only complete genomes as indicated in their NCBI record (dataset 1.1), resulting in a dataset with 1,777 sequences and 3,510 nucleotides in length from genotypes A–F. Dataset 1 was further subsampled for a genetic distance analysis (dataset 1.2), where 900 sequences consisting of complete genomes were used (see Methods subsection 2.4 for details).

The second dataset (dataset 2) was 3,182 nucleotides in length and included 93 sequences of genotype D sequences and Greenland sequences in order to assess the diversity of the novel sequences in a more granular way within genotype D, following the evidence from dataset 1 that these novel sequences fall within the genotype D clade. We kept two identical sequences which were serially collected from a single Greenland patient with Accession IDs JN792909 sampled in 1998, and JN792910 sampled in 2004. Out of the 39 Greenland sequences, 29 are novel and are published here for the first time (Supplementary Table 1). Seventeen sequences cover the full genome, while the remaining twelve are partial or nearly complete, though at least 1,253 nucleotides in length. We selected 54 sequences to represent HBV/D history and calibrate our data (Supplementary Table 1). Forty-two sequences serve as global representation of all subgenotypes (D1, *n* = 10; D2, *n* = 1; D3, *n* = 5; D4, *n* = 6; D5, *n* = 5; and D6, *n* = 5). An additional four are “novel quasi-subgenotype D2 of hepatitis B virus” full-length genome sequences from Taiwanese Indigenous peoples (Tran et al., 2014 AB555496, AB555497, AB555500, and AB555501), two are from a New Caledonia (HQ700511) and Argentina (JN688695) outgroup to the Indigenous taxa, and six are partial or nearly complete ancient HBV (aHBV) DNA sequences at least 943 nucleotides in length. Four aHBV sequences came from the Mühlemann et al. (2018) study (LT992438, LT992439, LT992444, and LT992454) and two aHBVs came from Krause-Kyora et al. (2018) study, a German medieval tooth and an Italian mummy available at PRJEB24921 of the European Nucleotide Archive. The ages of these ancient sequences ranged from 281 Before Common Era (BCE) to 1569 Common Era (CE).

The GenBank accession numbers for novel and previously published HBV genomes for dataset 2 are included in Supplementary Table 1.

### 2.3. Molecular Sequence Analyses

We computed multiple sequence alignments using MAFFT (Katoh and Standley, 2013) under default settings. We visualized the alignments and trimmed ragged edges with AliView (Larsson, 2014). We screened our datasets for recombination using RDP4 (Martin et al., 2015) applying six algorithms: Geneconv, Bootscan, MaxChi, Chimera, SiScan, and 3Seq.

The dataset 2 nucleotide alignment was translated to specific open reading frames for the polymerase and preS2 coding regions to determine the presence of signature amino acids associated with HBV/D subgenotypes within the Greenland sequences. We evaluated the amino acid residues for positions 39 (Pre-S2), 100 and 128 (POL Spacer), and 126 (RT) for all Greenland sequences and compared the observed residues to the predominant amino acids of subgenotypes D1 and D2, according to Yousif and Kramvis (2013). In addition, we mapped three distinct nucleotide mutations G1896A, A1762T, and G1764A across all Greenland taxa, as these are important markers associated with disease prognosis.

### 2.4. Genetic Distance

To calculate the genetic distance between the Greenland clade and other subgenotypes we estimated the evolutionary divergence over full-length genome sequence between groups as implemented in MEGA X (Kumar et al., 2016, 2018). The analyses were conducted using the Maximum Composite Likelihood model (Tamura et al., 2004). This analysis involved 900 nucleotide sequences from subgenotypes D1 (579), D2 (293), and Greenland (28) from dataset 1.2 (see Supplementary Material). Codon positions included were 1st + 2nd + 3rd + Non-coding. All ambiguous positions were removed for each sequence pair (pairwise deletion option). There were a total of 3,353 positions in the final dataset.

### 2.5. Phylogenetic Tree Search

To assign the novel HBV sequences from Greenland to the correct genotype we conducted a phylogenetic tree search on dataset 1 under the optimality criterion of maximum likelihood with substitution model testing as available in IQ-TREE (Nguyen et al., 2015). We executed a 1,000-replicate SH-aLRT measure of support (Guindon et al., 2010). “*The SH-aLRT is an approximation of the likelihood ratio, a direct measure of how much the evidence supports the hypothesis. SH-aLRT is an approximation of the ratio of the log-likelihood of the optimal hypothesis and the best contradictory hypothesis*” (Schneider et al., 2020).

In order to avoid any artifact or bias in the analysis, we tested dataset 1 against its subset dataset 1.1, and no major changes of topology were found. We also tested dataset 2 of the novel Greenland taxa against two subsets of it: (1) only full genomic sequences; (2) only full genomic sequences and unique individuals (oldest sequence from each individual was selected). We built a total of three ML trees using IQ-Tree and compared them. Neither subset tree had a change in the overall tree topology or affected the monophyly of the Greenland clade.

We used BEAST v1.10.4 (Suchard et al., 2018) to calculate the TMRCA of the Greenland taxa on dataset 2 (genotype D). Based on the partial fragmentation of our alignment (see Supplementary Material), we elected to treat the alignment as a single substitution partition, contrary to the eight-partition approach used on genotype B by Bouckaert et al. (2017). We selected the GTR+Γ_4_ substitution model with four empirical base frequencies, a Skyride coalescent tree prior (Minin et al., 2008), and an uncorrelated relaxed lognormal clock (URL). We also tested a strict clock; however, we did not observe convergence in Tracer (Rambaut et al., 2018), confirming the URL clock as the appropriate selection. We ran the model in duplicate with a Markov chain Monte Carlo length of 100 million. Convergence was determined by Effective Sample Sizes of at least 200 per statistic. The final maximum clade credibility tree with metadata annotation was rendered using FigTree (Rambaut, 2012).

## 3. Results

### 3.1. Maximum Likelihood Tree Indicates Greenland Lineage Independence

**Figure 2:**
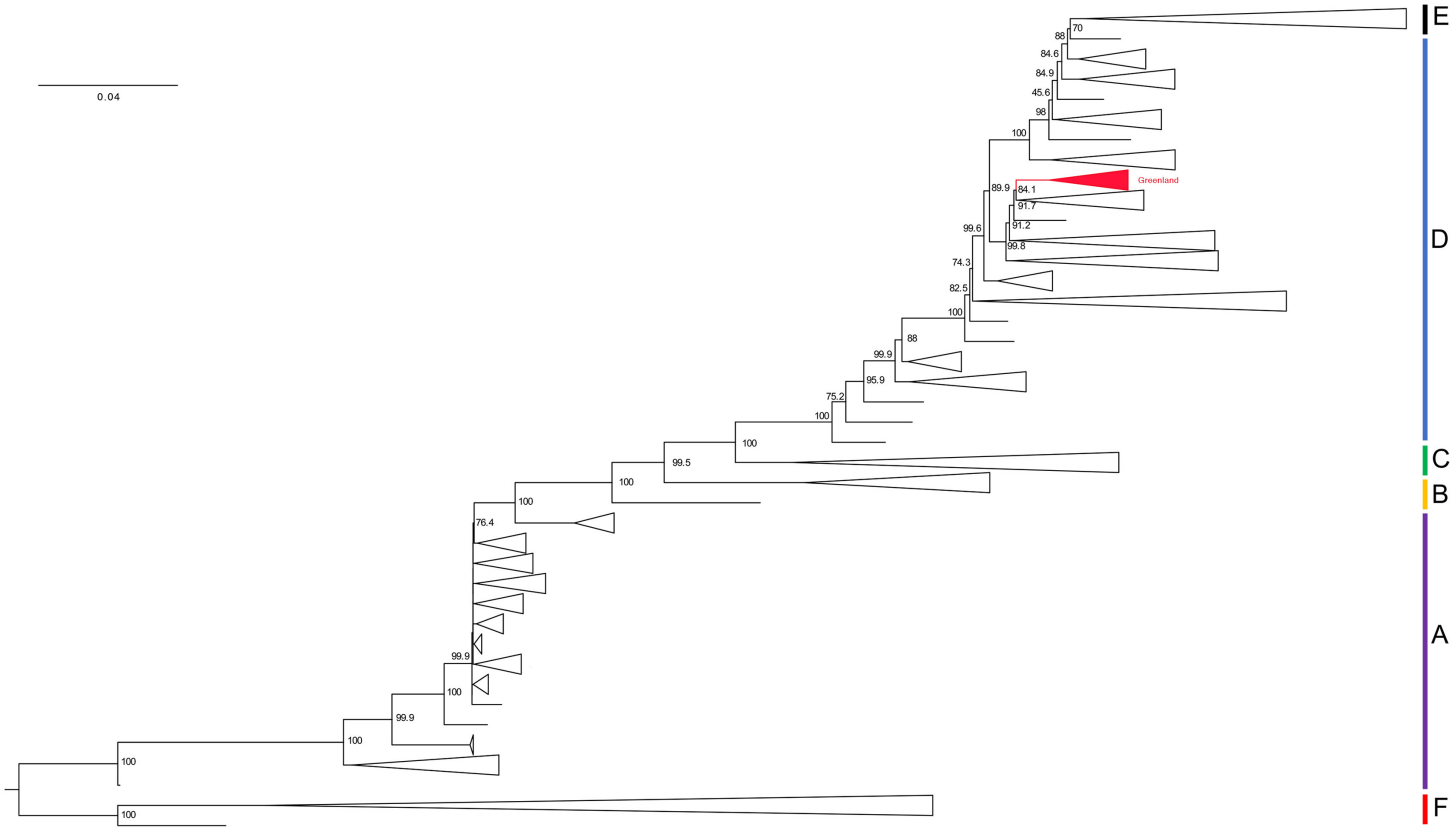
Phylogenetic hypothesis of dataset 1.1. Maximum-likelihood tree. Branch lengths represent an estimation of the average number of nucleotide substitutions per site. Node labels indicate SH-aLRT support. Clade names correlate to their genotype assignment and are represented by the vertical bars on the right of the tree. Red collapsed clade represents the Greenland taxa. See Supplementary Material for a full version of this tree.

Model testing prior to IQ-TREE building identified the best fit model from Akaike and Bayesian Information Criterion as GTR + F + R10. The maximum likelihood tree from dataset 1.1 has the Greenland taxa in a monophyletic group sister to genotype D sequences with 89.9% SH-aLRT-like support (Figure 2). Thus, we assigned them to genotype D for our downstream analyses. All subsequent references to our data refer to dataset 2 containing only genotype D sequences.

### 3.2. Recombination

We excluded no taxa based on recombination. RDP identified two potential recombinants (see Supplementary Material). The first we interpret to be false identification of the recombinant region from nucleotide position 1720–2579 in 11 D4/D6 sequences containing an 859 base pair section from D2 sequence AB267090. Based on the location of the areas internal and external to the recombination site in UPGMA trees, we see no evidence for inter-subgenotype recombination. An additional sequence, JN792912, which was labeled as mostly recombinant with unknown origin, was removed in addition to the eleven sequences and RDP was re-run. The same region was identified in all D3 sequences instead of D4/D6 with a supposed D1 parent. We hypothesize this is an artifact given the high nucleotide conservation of this region. The UPGMA tree topologies were not suspicious of recombinant template-switching in these taxa.

The second recombinant region flagged by RDP was in an Indian D5 sequence, GQ205382, with a 269 base pair region of a D3 sequence (KX827292) from nucleotides 1765 to 2034. This region has no mutations between the two sequences. Instead, the D3-specific single nucleotide polymorphisms (SNPs) are found in this D5 sequence, potentially representing a recombination event that cannot be excluded. However, the D5 sequence still clusters with other Indian D5 sequences in a clade with 100% SH-aLRT-like support in the ML tree. Neither of these taxa produced interference on ML tree topology. Thus, we elected to keep all of the 93 taxa. We found no recombination between D1, D2, and the Greenland sequences.

### 3.3. Amino Acid Signatures

The Greenland HBV/D amino acid signatures on Pre-S2 aa39, POL Spacer aa100 and aa128, and RT aa126 introduces a paradigm in relation to D1 and D2 signatures (Table 1). The Greenland sequences do not “fit” into either D1 or D2 subgenotypes amino acid signatures predominantly observed with these subgenotype sequences according to Yousif and Kramvis (2013). Instead, they share characteristics of D2 at PreS2 aa39 and POL aa100, and D1 at RT aa126 and POL aa128.

**Table 1:** Amino acid signatures of HBV subgenotype D1, D2 and Greenland taxa.

**AA Signature**	**D1**	**D2**	**Greenland**
Pre-S2 aa39	V/a	A/v	A
POL Spacer aa100	S/a, t	A/t,v	A
POL Spacer aa128	S/g	G/s	S
RT aa126	H	R/h	H

*Adapted from Yousif and Kramvis (2013)*.

### 3.4. Mutations

We observed mutations in our sequences at positions 1762 (wild type A; mutant T), 1764 (wild type G; mutant A), and 1896 (wild type G; mutant A). Twenty two of the 39 Greenland sequences had a G to A mutation at nucleotide position 1896. Of the 13 consecutively sampled taxa, only two pairs reflected this substitution, whereas most (*n* = 5) had a mutant A at position 1896 in both samples. Mutations A1762T and G1764A were not as prevalent, present in only 9/39 and 14/39 Greenland sequences, respectively (Table 2).

**Table 2:** Greenland HBV/D taxa and consecutive paired sera.

**Patient ID**	**Taxon ID(s)**	**Collection date**	**Community**
01	MT603386†_*nodata*_	2017	Aasiaat
02	MT603402	2004	Itilleq
03	JN792909; JN792910	1998; 2004	Itilleq
04	JN792911†,^•^,*; JN792912†,^•^,*; MT603404^•^,*	1998; 2004; 2008	Itilleq
05	MT603401; MT603403	2004; 2009	Itilleq
06	MT603400; MT603385	2004; 2009	Itilleq
07	MT603383†,^•^,*; MT603384†,^•^,*	2004; 2009	Itilleq
08	MT603382†,*_mixed_	2017	Nuuk
09	MT603376†	2017	Nuuk
10	MT603378†_*nodata*_	2017	Nuuk
11	MT603396†_*mixed*_	2017	Nuuk
12	JN792904†; JN792903	1998; 2004	Sarfannguaq
13	JN792908†; JN792907	1998; 2004	Sarfannguaq
14	JN792906†; JN792905†	1998; 2004	Sarfannguaq
15	MT603397†	1998	Sisimiut
16	MT603399†	2017	Sisimiut
17	MT603380†_*mixed*_	2017	Sisimiut
18	MT603381†,*	2017	Sisimiut
19	MT603377†,^•^_*mixed*_	2017	Sisimiut
20	MT603379†_*mixed*_	2017	Sisimiut
21	MT603392†,*; MT603389†,*	1998; 2008	Sisimiut
22	MT603391†; MT603390†	1998; 2008	Sisimiut
23	MT603388†,^•^,*; MT603387†,^•^,*	1998; 2008	Sisimiut
24	MT603398^•^,*; MT603393†,^•^,*	1998; 2008	Sisimiut
25	MT603394*; MT603395†,*	1998; 2008	Sisimiut

*Paired sera is indicated by multiple taxon IDs in a single cell separated by semicolon. Significant mutant nucleotides (bolded) are indicated by symbol, opposed to unmarked wild type nucleotides*.

† *= G1896A*,

• *= A1762T*,

* *= G1764A*.

### 3.5. Genetic Distance

The Greenland clade had an estimated evolutionary divergence of ~3% with both subgenotypes D1 and D2. Both D1 and D2 also shown a divergence of ~3% between each other (Table 3). We used the pool of complete genome sequences of the Greenland clade and compared to the pool of D1 and D2 sequences belonging to sister clades on the ML tree.

**Table 3:** Estimates of evolutionary divergence over sequence pairs between Greenland, D1 and D2 clades.

	**Greenland**	**D1**	**D2**
**Greenland**	0	0.0309	0.0322
**D1**	0.0309	0	0.0303
**D2**	0.0322	0.0303	0

*The number of base substitutions per site from averaging over all sequence pairs between groups are shown*.

### 3.6. Substitution Rate and TMRCA of HBV/D in Greenland Populations

We inferred on the molecular clock analysis a substitution rate of 1.398 × 10^−5^ [95% HPD 8.837 × 10^−6^, 1.952 × 10^−5^], similar to that obtained by Mühlemann et al. (2018): 1.18 × 10^−5^ [9.21 × 10^−6^, −1.45 × 10^−5^]. The age of the root, representing genotype D, was 675 BCE (1221 BCE, 281 BCE). The age of the Greenland taxa was 629 CE (95% HPD interval 37 CE, 1138 CE).

## 4. Discussion

Studies on HBV phylogenetics provide a means of understanding the history of the disease spread in an epidemiological context. In this study, we reveal a novel quasi-subgenotype of HBV genotype D isolated from Greenland. Interestingly, this novel clade, sister to subgenotype D2, shares genetic characteristics of both subgenotypes D1 and D2 and shares slightly reduced genetic divergence with subgenotype D1 (3.1%) compared to subgenotype D2 (3.2%) based on our data. Inference of all Greenland taxa as monophyletic in maximum likelihood and Bayesian frameworks supports the hypothesis of a single origin event giving rise to a distinct and isolated lineage on the west coast of Greenland.

Phylogeography in the Greenland dataset was not used given the limited geographic scope of the Greenland taxa and lack of logistical purpose exploring global movement given our reductive representation of genotype D taxa. The 1762 and 1764 site mutations are associated with a significantly increased risk of liver disease progression and hepatocellular carcinoma development (Yang et al., 2016; Lin and Kao, 2017). The mutation on site 1896 is associated with HBeAg negativity and seroconversion to anti-HBeAg positivity by introducing a stop codon to the HBeAg reading frame (Kramvis et al., 2018). Twenty two of the Greenlandic sequences shared a non-exclusive G to A substitution at nucleotide position 1896, associated with seroconversion of positive HBeAg reactivity to anti-HBeAg positivity and historically most present in genotype D (Zhand et al., 2015). These taxa likely had higher rates of evolution (Kramvis et al., 2018), though our methods were unable to tease apart their contribution to the substitution rate. Of our consecutively sampled pairs/triplet, the lack of precore mutation (*n* = 3) or substitution from the mutant to the wild type (*n* = 3) indicates active infection occurring over multiple years, though our lack of sampling only offers sporadic glances into infection dynamics. The identical pair of consecutively sampled sera separated by 6 years is interesting, as we expected this individual to seroconvert over this period. These findings reflect the slow and tangled nature of HBV evolution.

As the Greenland sequences are monophyletic, this suggests the presence of a lineage with a new amino acid signature. For instance, though no recombination was inferred between the Greenland sequences and subgenotype D1/D2, the overlapping serotype signatures (Table 1), suggest that the Greenland taxa cannot be neatly fit into the amino acid composition of either subgenotype.

**Figure 3:**
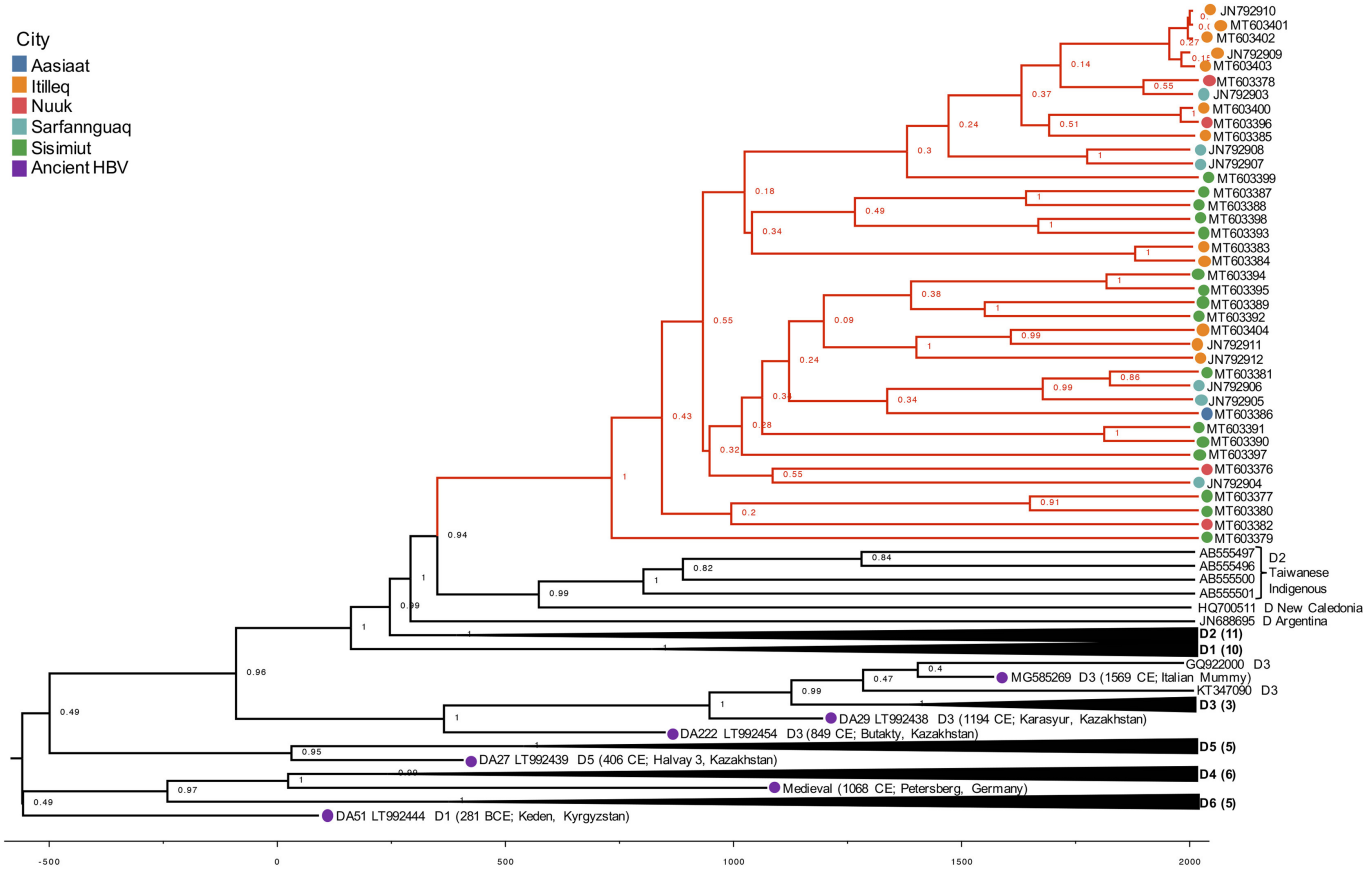
Phylogenetic hypothesis of dataset 2. HBV/D Maximum Clade Credibility (MCC) tree from combined run trees with posterior support indicated on nodes. Greenland lineages are shaded in red and corresponding location indicated by colored circle on branch tips. X-axis represents the age of nodes.

It is a well-known observation that subgenotype D1 shows intergroup divergence with D2 <4–7.5% divergence agreed upon as the definition of an HBV subgenotype (Yousif and Kramvis, 2013; McNaughton et al., 2020). The justification for classifying D1 and D2 as subgenotypes is based upon well-resolved phylogenetic clustering, supported by high bootstrap values (>85–90%), different serological subtypes, and different geographic distribution (Yousif and Kramvis, 2013). As observed in this study, the Greenland HBV clade is well-resolved and clusters completely separately with high confidence by aLRT-SH and posterior probability, and is unique to the geographic region of Greenland. However, in keeping with the suggestion of McNaughton et al. (2020), the Greenland clade may be considered a quasi-subgenotype due to its monophyletic clustering following maximum-likelihood analysis but with a genetic distance separating it from D2 and D1 of ~3%. Based on tree topology, this quasi-subgenotype clusters most closely with HBV subgenotype D2. The Greenland taxa's inferred sister clade, which includes HBV subgenotype D2 from Taiwanese Indigenous Peoples, New Caledonia, and Argentina, suggests a single origin between the Greenland and D2 taxa that is not inclusive of D1, with 94% posterior support (Figure 3).

Taxa are likely to be geographically related, preserving the complication that may be faced with pure geographic assignment (de Bernardi Schneider et al., 2019; Mojsiejczuk et al., 2020). Rather than focusing on endemicity, location, or solely genetic distance, the best way to characterize HBV may be by observing evolutionarily independent lineages through phylogenetic analysis, as is argued by Peterson (2014). Phylogenetic analysis circumvents the use of genetic distance as a sole metric for sub-/geno-type naming. Further, while genetic distances between the Greenland taxa and subgenotypes D1 and D2 (3.1 and 3.2%, respectively) are within what McNaughton et al. (2020) cites as the majority of genotype D pairwise distance (i.e., 2–4%, compared to the typical subgenotype divergence of 3–8%), they are also distinctly monophyletic across our methods. Here, we present our inference of 39 Greenland HBV/D taxa as a newly characterized quasi-subgenotype exemplified by this topologically independent lineage.

The Greenland taxa's TMRCA of 629 CE translates to a coalescence point about 1,390 years before present (YBP), preceding the only other Greenland HBV lineage (B5) studied (Bouckaert et al., 2017). Their study estimated that B5 was introduced to Greenland during coastal route movement of the Thule in the last 1,000 years (Bouckaert et al., 2017), calibrated by the date associated with Thule migration to the Eastern Arctic (647–953 YBP or 1370–1064 CE). Nonetheless, they are distinct genotypes evolving at different rates, thus it is feasible that these genotypes arrived in Western Greenland following separate introductions.

The more closely related subgenotype D2 may have an origin from the Middle East (Kostaki et al., 2018) or Southern Europe (Spitz et al., 2019). The use of ancient HBV DNA allowed us to estimate a Greenland HBV/D TMRCA range of 37–1138 CE. Similar to what has been described with HBV genotype B-infected indigenous people in the circumpolar Arctic (Bouckaert et al., 2017), this result suggests that the virus was introduced at some point to the Thule, but this ancestor no longer exists. However, ancestral remnants aside from the Taiwanese Indigenous and New Caledonian samples which formed an adjacent clade to the Greenland taxa are necessary for improved confidence in inferring the route which brought HBV/D to these populations.

Integrating novel sequences with published HBV/D data in this study has demonstrated a strongly inferred and geographically independent monophyletic lineage from existing HBV/D subgenotype architecture. We reveal that HBV/D samples from 25 Western Greenland residents, 13 of whom were consecutively sampled over 5–10 years, form an evolutionarily unique clade distinct from subgenotypes D1 and D2.

Pairing these modern data with ancient DNA, we uncovered more detail in the story of HBV genotype D evolution. Such diachronic studies are necessary for the tough-to-interpret HBV (Mühlemann et al., 2018), where study results on rate and origin vary by orders of magnitude subjective to data and model limitations. Additional HBV/D sequences from ancient archeological remains in the Arctic are necessary to resolve the mystery of its origin and pattern of dispersal beyond speculation.

## Data Availability Statement

The taxa utilized in this study can be found in Supplementary Table 1. All supplementary data, which includes all trees from this study and metadata, can be found at GitHub (https://github.com/abschneider/Paper_HBV_Greenland).

## Ethics Statement

The studies involving human participants were reviewed and approved by the Statens Serum Institute (Copenhagen, Denmark) and the Commission for Scientific Research in Greenland (Approval number 505-99). Written informed consent was obtained from each participant. The patients/participants provided their written informed consent to participate in this study.

## Author Contributions

HK and MB contributed to the acquisition of study samples. AB, CO, and RH contributed substantially to the conception and design of the work and the drafting and critical revision of the manuscript. AB, CO, and JW contributed to the revised version of the manuscript. AB, RH, HK, MB, YT, TM, CO, and JW contributed to the analysis and interpretation of data and manuscript revision. All authors contributed to the article and approved the submitted version.

## Conflict of Interest

The authors declare that the research was conducted in the absence of any commercial or financial relationships that could be construed as a potential conflict of interest.
