# Supplementary material for: Analysis of Hepatitis B Virus Genotype D in Greenland Suggests the Presence of a Novel Quasi-Subgenotype
Source: Front Microbiol. 2021 Jan 15;11:602296. doi: 10.3389/fmicb.2020.602296 (PMC7843931; doi:10.3389/fmicb.2020.602296)
Supplement: Supplementary file 1 [file Data_Sheet_1.PDF]

frontiers

Supplementary Material

Table S1: Metadata for study taxa. *Dates were calculated by subtracting "years before present" value in each taxon name from 2016, the date of GenBank reference database download.

Taxon ID	Accession ID	Subgenotype	Date	Location
208	MT603390	D	2008.21	Sisimiut, Greenland
214	MT603389	D	2008.21	Sisimiut, Greenland
218	MT603404	D	2008.21	Itilleq, Greenland
248	MT603395	D	2008.21	Sisimiut, Greenland
267	MT603393	D	2008.21	Sisimiut, Greenland
268	MT603387	D	2008.21	Sisimiut, Greenland
345	JN792905	D	2004.96	Sarfannguaq, Greenland
417	MT603400	D	2004.96	Itilleq, Greenland
422	MT603383	D	2004.96	Itilleq, Greenland
437	MT603402	D	2004.96	Itilleq, Greenland
449	JN792912	D	2004.96	Itilleq, Greenland
473	JN792907	D	2004.96	Sarfannguaq, Greenland
1205	JN792909	D	1998.87	Itilleq, Greenland
1509	MT603385	D	2009.71	Itilleq, Greenland
1776	MT603391	D	1998.87	Sisimiut, Greenland
2031	MT603388	D	1998.87	Sisimiut, Greenland
2132	MT603398	D	1998.87	Sisimiut, Greenland
2335	MT603394	D	1998.87	Sisimiut, Greenland
2903	MT603397	D	1998.87	Sisimiut, Greenland
2943	JN792904	D	1998.87	Sarfannguaq, Greenland
2951	JN792908	D	1998.87	Sarfannguaq, Greenland
2952	JN792911	D	1998.87	Itilleq, Greenland
2958	JN792906	D	1998.87	Sarfannguaq, Greenland
3288	MT603392	D	1998.87	Sisimiut, Greenland
5180	MT603403	D	2009.46	Itilleq, Greenland
5198	MT603384	D	2009.46	Itilleq, Greenland
30127	JN792903	D	2004.96	Sarfannguaq, Greenland
302000479	MT603401	D	2004.96	Itilleq, Greenland
312000479	JN792910	D	2004.96	Itilleq, Greenland
101102-700441	MT603399	D	2017.50	Sisimiut, Greenland
101105-706980	MT603380	D	2017.50	Sisimiut, Greenland
101116-458966	MT603381	D	2017.50	Sisimiut, Greenland
101138-639190	MT603382	D	2017.50	Nuuk, Greenland
101148-137251	MT603386	D	2017.50	Aasiaat, Greenland
101148-241032	MT603376	D	2017.50	Nuuk, Greenland
101156-155760	MT603378	D	2017.50	Nuuk, Greenland

Table S1: Continued metadata.

Taxon ID	Accession ID	Subgenotype	Date	Location
101161-498572	MT603396	D	2017.50	Nuuk, Greenland
101192-645410	MT603377	D	2017.50	Sisimiut, Greenland
101195-133160	MT603379	D	2017.50	Sisimiut, Greenland
aHBV	ERS2200178	D	1068	Petersberg, Germany
aHBV-DA222	LT992454	D3	849*	Butakty, Kazakhstan
aHBV-DA27	LT992439	D5	406*	Halvay3, Kazakhstan
aHBV-DA29	LT992438	D3	1194*	Karasyur, Kazakhstan
aHBV-DA51	LT992444	D1	-281*	Keden, Kyrgyzstan
aHBV-NASD24SEQ	MG585269	D3	1569	Italy
N/A	GQ205384	D5	2008.13	India
N/A	GQ205382	D5	2008.04	India
N/A	KP322603	D5	2013.88	India
N/A	GQ205377	D5	2007.20	India
N/A	GQ205387	D5	2008.79	India
N/A	JN688695	D2	2009.50	Argentina
N/A	GQ922003	D4	1984.88	Northwest Territories
N/A	KU736923	D6	2006.96	Somalia
N/A	KP168419	D6	2012.04	Kenya
N/A	HQ700536	D4	2004.04	Kiribati
N/A	HQ700501	D4	1991.04	Samoa
N/A	HQ700525	D4	2004.04	Papua New Guinea
N/A	KNA827299	D6	2010.13	USA
N/A	KJ470884	D4	2010.20	Brazil
N/A	KP322604	D6	2013.88	Tunisia
N/A	KU736921	D6	2011.54	Ethiopia
N/A	FJ692533	D4	2006.04	Haiti
N/A	GQ477453	D2	2006.20	Poland
N/A	AB555500	D2	2009.50	Taiwanese aborigines
N/A	AB555497	D2	2009.50	Taiwanese aborigines
N/A	AB555496	D2	2009.50	Taiwanese aborigines
N/A	KJ647352	D2	2011.20	Argentina
N/A	AB555501	D2	2009.50	Taiwanese aborigines
N/A	FJ349205	D2	2008.96	Belgium
N/A	JQ687532	D2	2008.13	Serbia
N/A	GQ924652	D2	2007.04	Malaysia
N/A	FJ349220	D1	1999.88	Belgium
N/A	FJ349218	D2	1999.54	Belgium
N/A	HQ700511	D	2001.50	New Caledonia
N/A	AB267090	D2	2005.71	Japan
N/A	GQ477459	D1	2006.20	Poland
N/A	KF584162	D1	2012.20	Argentina
N/A	KF584161	D1	2011.29	Argentina

Table S1: Continued metadata.

Taxon ID	Accession ID	Subgenotype	Date	Location
N/A	KF061168	D1	2012.29	Pakistan
N/A	KU736925	D2	2008.88	Sudan
N/A	KP322601	D2	2013.88	India
N/A	JNA096956	D2	2006.37	Latvia
N/A	KNA827301	D2	2010.13	USA
N/A	JN257193	D1	2009.13	Syria
N/A	FJ904443	D1	2005.96	Tunisia
N/A	GQ377589	D1	2007.20	China
N/A	KP322599	D1	2013.88	India
N/A	JN257185	D1	2008.71	Syria
N/A	GQ922000	D3	1985.04	Northwest Territories
N/A	KP090177	D3	2003.46	Brazil
N/A	FJ349209	D3	1998.29	Belgium
N/A	KNA827292	D3	2010.13	USA
N/A	KT347090	D3	2007.04	South Africa
